# Supplementary material for: MetaRibo-Seq measures translation in microbiomes
Source: Nat Commun. 2020 Jun 29;11:3268. doi: 10.1038/s41467-020-17081-z (PMC7324362; doi:10.1038/s41467-020-17081-z)
Supplement: Supplementary file 10 — Supplementary Data 7 [file 41467_2020_17081_MOESM10_ESM.zip › File2/Confidence_High_Taxonomy/338398_out.krona.html]

Javascript must be enabled to view this page.

members
magnitude
magnitudeUnassigned
count
unassigned
taxon
rank

338398\_out

38

superkingdom
38
2

1239
38
phylum

1263003
18
species

SRS023914\_contig\_number\_21064SRS049959\_contig\_number\_18475SRS052697\_contig\_number\_37071SRS054905\_contig\_number\_2893SRS055017\_contig\_number\_5509SRS075078\_contig\_number\_19314SRS075773\_contig\_number\_21516SRS075821\_contig\_number\_22200SRS075984\_contig\_number\_14797SRS1041145\_contig\_number\_34002SRS104400\_contig\_number\_20323SRS142923\_contig\_number\_19619SRS144537\_contig\_number\_contig-100\_4942.271438SRS148817\_contig\_number\_13871SRS893187\_contig\_number\_3465SRS893383\_contig\_number\_34801SRS971275\_contig\_number\_contig-100\_11253.202212SRS971276\_contig\_number\_contig-100\_1786.192658

20
186801
class

20
186802
order

1
216572
family

genus
1
459786


SRS078242\_contig\_number\_20492
species
1
876091

1
1898207

SRS047433\_contig\_number\_10679
species


SRS148424\_contig\_number\_33917
species
1950886
1


SRS042966\_contig\_number\_16434SRS045645\_contig\_number\_9015SRS050752\_contig\_number\_19470SRS064757\_contig\_number\_18167SRS075341\_contig\_number\_19661SRS077024\_contig\_number\_13777SRS077392\_contig\_number\_22461SRS1041039\_contig\_number\_25496SRS1041133\_contig\_number\_16871SRS1041137\_contig\_number\_11048SRS1041139\_contig\_number\_3102SRS143466\_contig\_number\_2137SRS143895\_contig\_number\_13232SRS147557\_contig\_number\_17405SRS148196\_contig\_number\_27648SRS892882\_contig\_number\_contig-100\_1596.43834SRS893366\_contig\_number\_19558
species
17
172733
